# The potential therapeutic impact of a topical bacteriophage preparation in treating *Pseudomonas aeruginosa*-infected burn wounds in mice

**DOI:** 10.1016/j.heliyon.2023.e18246

**Published:** 2023-07-13

**Authors:** Hanieh Piranaghl, Shiva Golmohammadzadeh, Vahid Soheili, Zahra Sabeti Noghabi, Bahram Memar, Seyede Melika Jalali, Zhila Taherzadeh, Bibi Sedigheh Fazly Bazzaz

**Affiliations:** a Pharmaceutical Control Department, School of Pharmacy, Mashhad University of Medical Sciences, Mashhad, Iran; b Nanotechnology Research Center, Institute of Pharmaceutical Technology, Mashhad University of Medical Sciences, Mashhad, Iran; c Department of Biopathology, Faculty of Veterinary Medicine, Ferdowsi University of Mashhad, Mashhad, Iran; d Department of Pathology, Faculty of Medicine, Mashhad University of Medical Sciences, Mashhad, Iran; e Targeted Drug Delivery Research Center, Mashhad University of Medical Sciences, Mashhad, Iran; f Biotechnology Research Center, Institute of Pharmaceutical Technology, Mashhad University of Medical Sciences, Mashhad, Iran

**Keywords:** Bacteriophage, Burned wounds, Mice, Ointment, Polyethylene glycol, *Pseudomonas aeruginosa*

## Abstract

**Aim:**

This study compared a topical formulation containing lytic phages with a routine antibiotic in the murine model of burn/*Pseudomonas aeruginosa* infected wound healing.

**Methods & Materials:**

Isolated and purified lytic bacteriophages from hospital sewage were added to the polyethylene glycol (PEG) based ointment. A second-degree burned wound on the back of twenty-four adult female mice was created. The wounds were infected subcutaneously with 100 μL of 1 × 10^2−3^ CFU/mL *P. aeruginosa*. After 24 h, mice were randomly assigned to one of four groups: mice received a standard antibiotic (antibiotic-treated group), mice received an ointment without bacteriophage (PEG-based group), mice received a PEG-ointment with bacteriophage (bacteriophage-treated group), or mice received no treatment (untreated-control group). Every two days, the contraction of burned wounds, physical activity, and rectal body temperature were recorded. On day 10, mice were sacrificed, and the wounds were cut off and evaluated histopathologically.

**Results:**

In ointments containing PEG, bacteriophages were active and stable. The mice receiving bacteriophage and PEG-based ointment had substantially different wound contraction in primary wound healing (*P* = 0.001). When compared to the control group, the bacteriophage-treated group showed significant variations in wound contraction (*P* = 0.001). The wound contraction changed significantly between the antibiotic and PEG-based groups (*P* = 0.002). In all groups, physical activity in mice improved over time, with significant differences (*P* = 0.001). When the 8th day was compared to the days 2, 4, and 6, significant changes were found (*P* = 0.001, *P* = 0.02, and *P* = 0.02, respectively). Both the positive control and bacteriophage-treated groups showed perfect wound healing histopathologically. However, no significant variations in microscopic histopathological criteria were found between the groups.

**Conclusion:**

Formulated phage ointment could be a promising approach for treating infected burn wounds infected by *P. aeruginosa* in mice with no allergic reactions.

## 1 Introduction

Now, infections of burned wounds have become a major issue in patients with third-degree burns, and the chance of survival is determined by the seriousness of the burn and the post-burn infections [1]. People with burn injuries usually need to remain hospitalized for an extended period; they are at risk of post-burn infections caused by pathogens that are resistant to multiple drugs. The destruction of the stratum corneum layer in burned wounds leads to immunosuppression and post-microbial infections, making it the greatest cause of death for burned patients. The burned wound offers a substantial, nourishing environment comprising necrotic tissues and protein-rich wound exudate, promoting bacterial growth [2,3]. The wound healing process is affected by a limited blood supply in the burn area, making many delivery routes less effective [1]. Wound from burning are susceptible to infection, and *Pseudomonas aeruginosa* is a common pathogen linked to these injuries.

*P. aeruginosa* is a Gram-negative organism that takes advantage of oxygen-rich environments to cause illness in humans and animals. It is the primary source of numerous intricate illnesses, including ear and eye diseases, stubborn post-burn infections, and skin grafts that were declined [4]. The most effective antibiotics have no effect on *P. aeruginosa* and it quickly develops new ways to resist them.

Because of the emergence of multidrug-resistant bacteria species that cause post-burn infections, it is imperative to seek alternative treatments. The most ancient and pervasive entities on the earth, bacteriophages, are tiny viruses that measure between 20 and 200 nm. These microorganisms possess a power that allows them to damage their host bacteria while leaving human or animal cells unscathed. Antibiotics can be substituted with bacteriophage therapy [5,6], or used together [7]. Bacteriophages can be divided into two categories based on the way they infect. Lytic phages are in the first group, while the second group has lysogenic phages. The suitable type of bacteriophage for bacteriophage therapy is the Lytic phage [8]. Bacteriophages have several advantages over traditional antibiotics, including their specificity for certain types of bacteria, their ability to grow along with their bacterial hosts, and their ability to penetrate biofilms, which are often a barrier to antibiotic penetration. However, antibiotic therapy showed more resistant and side effects [9]. A few studies have shown promising results in animal models, where bacteriophages were effective in treating infected wounds and reducing bacterial load [[10], [11], [12], [13], [14]]. There are different reports on the therapeutic use of phage cocktails for controlling various bacterial infections [15,16]. Phage combinations appear preferable to single phages when preventing the creation of resistant mutants. Phage mixtures may be a viable therapeutic alternative when antibiotic therapy cannot relieve bacteria-mediated burn wound infections [15]. It is important to note that the use of bacteriophages as a therapeutic agent is still in its early stages and is not yet widely available as a standard treatment option.

Semi-solid preparations are commonly used for topical application on wounds, as they offer a physical barrier and can provide a moist environment for wound healing [[17], [18], [19]]. There is evidence to suggest that PEG-based semi-solid preparations may be effective in treating burn wounds infected with *P. aeruginosa* [20,21]. It has been showed that this preparation could be a useful alternative to traditional burn wound dressings for treating infections caused by *P. aeruginosa.*

We innovated by adding a mixture of phages to polyethylene glycol (PEG) ointment base and evaluating its therapeutic potential in mice with burns and wounds infected by *P. aeruginosa.* We used a standard bacterial cell (*P. aeruginosa*) from Persian Type Culture Collection, Iran (PTCC 1074) and bacteria-specific phages from sewage.

## 2 Methods and materials

### 2.1 Phage isolation, purification, and propagation

Using a membrane filter (pore size 0.22 μm), we collected sewage samples from the third sewage pond in Ghaem Hospital in Mashhad. Then, we used these samples to isolate *P. aeruginosa-*specific phage [[22], [23], [24]].

Briefly, according to the literature [25], *P. aeruginosa* PTCC 1074 was inoculated into a test tube containing 5 mL of tryptic soy broth (TSB, HiMedia, India, and MgSO_4_ 0.001 M) and 2 mL of the filtrated sewage, and incubated overnight in a shaker incubator (37 °C, 100 rpm). Upon completion of the incubation period, the tube contents were moved to sterile centrifuge tubes and spun at 4500×g for 5 min. A membrane filter (0.22 μm) was used to sift the supernatant to get rid of any bacterial residue.

For plaque formation, we used the bilayer agar plate method. The base layer containing soft agar (Tryptic Soy Agar; TSA, HiMedia, India, with MgSO_4_ 0.001 M) was prepared. Following that, 100 μl of the bacteriophage filtered and 100 μl of a *P. aeruginosa* suspension that had been left overnight were blended with 2 mL of media (Tryptic Soy Broth; TSB, HiMedia, India, with 0.7% agar and MgSO_4_, 0.001 M) as top agar. Incubation of the plates was done for 24 h at 37 °C. For having a control, 100 μl of a *P. aeruginosa* overnight suspension was blended with 2 mL of top agar, put on a soft base agar plate, and left to incubate for 24 h at 37 °C [26]. After the incubation period elapsed, the plates were examined. The plates with bacteriophages had more transparency than the control plates. A transparent plate containing phages was harvested from the broth culture medium to provide the correct amount of bacteriophages, and it was serially diluted into 12 dilutions (10^−1^ to 10^−12^ PFU/mL). The bilayer method was employed to culture each dilution. A colony counter device (aCOLyte, Synbiosis, UK) was used to keep track of the number of phage plaques.

To collect more phages to use in the future, the plate surface was washed with the broth culture medium, and 1.8 mL of the phage suspension was combined with 10% glycerol, and placed in a freezer at −70 °C.

### 2.2 Bacteriophage host range/spot test

To investigate the host range of the isolated phages, spot tests were performed on five *P. aeruginosa* clinical isolates from the burn section of Ghaem Hospital (Mashhad, Iran), and one standard strain (*P. aeruginosa* PTCC 1074), following the method described by Ghasemi et al. [27] with some modifications.

Overnight of each bacterial culture (0.1 mL, 10^8^ CFU/mL) was mixed with 2 mL TSB (containing 0.7% agar, MgSO_4_ 0.001 M, at 45^○^C). Then the mixture as mentioned above was spread over the surface of the agar plate -TSA containing MgSO_4_ 0.001 M. Phage filtrate (10 μl, 10^8^ PFU/mL) was then spotted on the top agar layer of plates and incubated (37^○^C, overnight) after the top agar was hardened. Host cell lysis appeared as clear zones on the plates, showing the ability of the phage to infect the bacterial isolates tested [25,28].

### 2.3 Transmission electron microscopy of bacteriophage

Transmission electron microscopy was performed as previously described by Shahin and Bouzari [29]. The phage suspension (10^12^ PFU/mL) was concentrated by ultracentrifugation (25000×g, 120 min, Hanil, Ultra 5.0, South Korea). Pellet was carefully resuspended in distilled water after the supernatant was removed, and then 10 μl was put on a 400-mesh grid and negatively stained (2% uranyl acetate). The solution was drained with the filter paper, and the phage particles were inspected (electron microscope, Leo, Zeiss, Germany).

### 2.4 PEG ointment preparation and stability analysis

An ointment containing phage (PEG 400/PEG 4000 = 8:4 (g)) was formulated in a semi-solid state for primary treatment, and another formulation of PEG 400/PEG 4000 = 9:3 (g) without phage was used as a control. For every formulation, phages were combined with the base at a concentration of 10^7^ PFU/mL, and then mixed with a geometric dilution process in order to get a uniform mass.

To see how stable lytic phage is in PEG ointments, we used an agar lawn plate of *P. aeruginosa* PTCC 1074. Five dilutions of ointment were syringed onto the lawn's surface in order and incubated (37^○^C, 24 h). Distinct zones on the agar plate's surface showed the lytic capacity of phages in the ointment [30]. To assess the ointment's stability, after 0, 2, and 4 months, the ointments kept at 4^○^C, 20 ^○^C, and 40^○^C were characterized, and the pH changes were recorded [31].

### 2.5 Animals and primary supportive treatments

This research was approved by the Mashhad University of Medical Sciences Animal Experimentation Ethics Committee (IR.MUMS.PHARMACY.REC.1397.063) and was conducted with veterinary oversight and surveillance. All relevant international, national, and institutional guidelines for the welfare of animals were heeded.

For this study, 24 adult female mice with a weight of 25–30 g were chosen to be part of a controlled experimental design. The decision for this sample size was influenced by logistical constraints and ethical considerations, including animal welfare and limited resources.

The four experimental groups; antibiotic-treated group, bacteriophage-treated group, PEG-based group, and untreated-control group, were randomly populated with 6 mice each (the Animal House at the Mashhad University of Medical Sciences in Iran was the source of the animals). Animals were kept isolated in cages at a temperature of 22 ± 4 °C, 10% humidity, and a 12/12-h light/dark cycle, with open access to food and taped water.

An intraperitoneal injection of ketamine (100 mg/kg) and xylazine hydrochloride (10 mg/kg) was used to anesthetize the mice [32]. Sedated mice had their hair clipped off from the back and then shaved. Afterward, the mice were depilated on their backs so that a square wound could be made in that region.

A plastic template and electric kettle provided and conducted hot steam water to the back of the mice [33]. A 15 × 15 mm burned wound on the shaved back of the mice was created by having each mouse in direct contact with a conduction steam tube for 10 s [33]. Subsequently, each mouse was given 0.5 mL of dextrose saline serum intravenously to prevent hypovolemic shock. Acetaminophen, at a concentration of 0.25 mg/mL, was blended into the mice's drinking water to provide pain relief [34]. In 30 min, 100 μl of a 10^2^–10^3^ CFU/mL *P. aeruginosa* inoculum was injected subcutaneously to initiate infection [35]. In order to prevent contact with other mice's skin, each mouse was put in its own cage with a designated tank for food and water. The temperature of the room was kept at the right level for the animals. For the next three days, all animals were administered 75 IU of vitamin A, 20 IU of vitamin D3, and 0.5 mL of Dextrose/Saline serum as primary supportive treatments.

## 3 Main treatment protocols

A day following, each group of mice was given one of these treatments for ten days:

For Group I (the bacteriophage-treated group), mice were administered PEG base ointment (PEG 400/PEG 4000 in 8:4 ratio) containing 10^7^ PFU/mL bacteriophage and applied to their burned wounds with infection once daily.

Group II (positive control group, antibiotic-treated group): mice received a single daily application of silver sulfadiazine ointment to their infected burned wounds.

Group III (negative control group, untreated-control group): no treatment was administered to the mice.

For Group IV (PEG-based group), mice were given a PEG-based ointment (PEG 400/PEG 4000 = 9:3) that was free of bacteriophage once a day to determine whether the carrier ointment would affect the treatment process or not.

### 3.1 Morphological, clinical, and physical assessments

The wound contraction, rectal body temperature, and physical activity of the burned mice were assessed and documented every other day. To calculate the wound contraction as a percentage of the initial wound size, use this: [(day 0 area − day n area)/(day 0 area)] ×100%. A positive change shows a reduction in the burned wound's size, and a negative change shows an increase in the burned wound's size. The surface area of the wound (mm^2^) was assessed using Digimizer software and calculated through the Meeh formula [36]. The physical activity levels of the mice are detailed in Table 1 and rated using the Kumari et al. system [34].

**Table 1:** Scoring system to measure physical activity of mice.

Appearance characteristics	Score
Normal and unremarkable	5
Slight illness, lethargy, and ruffled fur	4
Moderate illness, severe lethargy, ruffled fur and hunched back	3
Severe illness, above signs plus exudative accumulation around partially closed eyes	2
Moribund state	1
Death	0

All groups had their visible skin damage and irritation monitored over 24 h (including progress of the rash, inflammation, swelling, scaling, and abnormal tissue growth) [[37], [38], [39]].

### 3.2 Histopathological assessments

On the 10th day, the mice were sacrificed by the cervical dislocation procedure. We excised the entire burned wound site and promptly fixed it in 4% formaldehyde (v/v) combined with PBS (0.01 M, pH 7.2). After that, we went through the usual histological steps, including dehydration in a sequence of alcohol, clearing with xylene, and finally embedding the tissue in paraffin at temperatures between 58 °C and 60 °C. To finish, we put forward a hematoxylin and eosin staining process. Histopathological evaluation and scoring were carried out according to the method described by Tkalcević et al. [38].

This is a description of granulation tissue:

i. Immature granulation tissue: macrophages, fibroblasts, and growing vessels within loose granulation tissue.
ii. Mature granulation tissue: fibroblasts and proteins from the extracellular matrix in a less dense arrangement from layers and vessels that are positioned perpendicularly.
iii. Fibrosis: granulation tissue is dominated by extracellular matrix proteins, mainly collagen, with fewer fibroblasts and vessels.

Each wound received a semiquantitative rating of 1 to 3 for each of the three granulated tissue covers:

1. Part of the wound bed is covered by granulation tissue.
2. The wound bed is covered by a thin layer of granulation tissue.
3. The entire wound bed was covered in thick granulation tissue.

This study also includes the following:

- Quantification of soft tissue necrosis (subcutaneous fat and skeletal muscle):
  1. Sever: over half of the subcutaneous soft tissues suffered from necrosis.
  2. Moderate: 10–50% of subcutaneous soft tissue suffered from necrosis.
  3. Minimal to mild: less than 10% of subcutaneous soft tissue suffered from necrosis.
- Inflammatory score
  1. Severe inflammation comprises extensive and condensed inflammatory cells
  2. Moderate inflammation comprises a few inflammatory cells
  3. Minimal to mild inflammation comprises sparse inflammatory cells
- Reepithelialization score:
  1. Incomplete
  2. Complete, thin epithelialization
  3. Complete, near-normal epithelialization

### 3.3 Chemicals

All chemical products were from Sigma-Aldrich Company (Sigma-Aldrich Chemical Co., Steinheim, Germany).

### 3.4 Statistical analyzes

Data difference analysis was handled by SPSS 25.0 (IBM, USA), and Graphpad 8.0 (USA) was used for data visualization. Fisher's exact test was used to compare proportions. One-way ANOVA was employed to examine the differences between groups at each time point. Repeated measurement analysis of variance (ANOVA) was used for all continuous variables. The comparison between multiple groups at different time points was evaluated using two-way repeated measures ANOVA with Tukey's multiple comparisons *post-hoc* test. All comparisons were two-sided tests. 95% was taken as the confidence interval and the difference was statistically significant when the P-value was less than 0.05. The data is expressed as mean ± SD. Each animal group comprised 6 animals.

## 4 Results

### 4.1 Plaque phage morphology and its enumeration

**Fig. 1:**
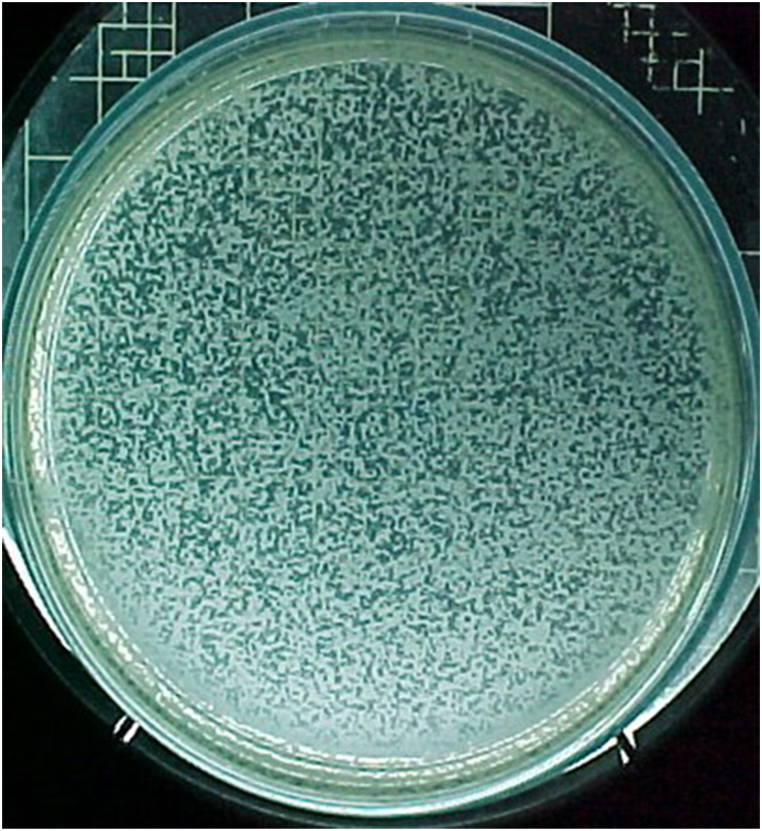
**Bacteriophage plaques on the soft agar subculture of *Pseudomonas aeruginous*.** A and B show the plaques (A (10^−9^ PFU), 10 times more diluted than B (10^−8^ PFU)). All the plaques of *Pseudomonas aeruginosa* bacteriophages are clear and circle with 0.5–1 mm diameter.

Unoccupied areas on the plate's surface corroborated that the bacteriophages were in the lytic phases (Fig. 1A), 10^−^^9^ PFU; All the plaques are clear and circle with 0.5–1 mm diameter.

Considering that 500 plaques were counted in 10^−9^ dilution (Fig. 1 A), and considering the volume of 100 μL phage dilution, the initial concentration of phage suspension was about 5 × 10^12^PFU/mL.

### 4.2 Bacteriophage host range/spot test

Spot test showed that only two strains containing standard strain (33%) were susceptible to the isolated phage, and the others (4 strains) were insensitive to this phage.

### 4.3 Transmission electron microscopy of bacteriophage

**Fig. 2:**
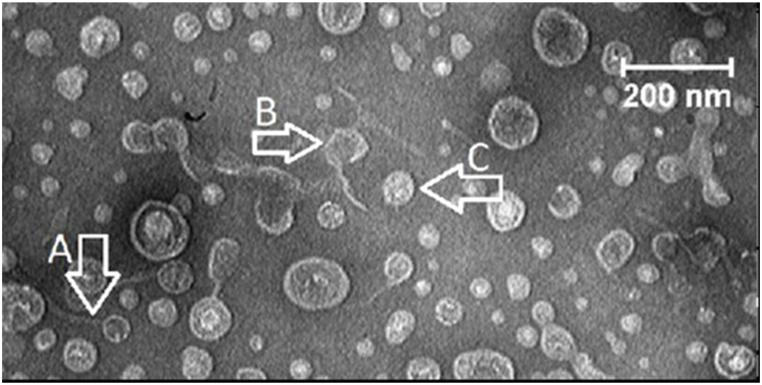
**Transmission electron micrographs.** Bacteriophages morphology as displayed by electron magnifying lens showing diverse morphotypes: The Siphoviridae phage with a head diameter of 59 nm and a tail of 189 nm (A), the Myoviridae family has a head diameter of 72 nm and an extended tail of 161 nm (B), the *Podoviridae* family all had isometric heads and a very short tail, not always visible in the electron microscope (C).

In multiphage samples, we observed ubiquitous tailed phages from the *Caudovirales* order, containing dsDNA that comprise the three families (Fig. 2), *Myoviridae*,*Siphoviridae*, and *Podoviridae,* with helical tail built of subunits [40]. The *Siphoviridae* phage (A in Fig. 2), the *Myoviridae* family (B in Fig. 2), and the *Podoviridae* family (C in Fig. 2).

### 4.4 PEG-based bacteriophage ointment and stability analysis

**Fig. 3:**
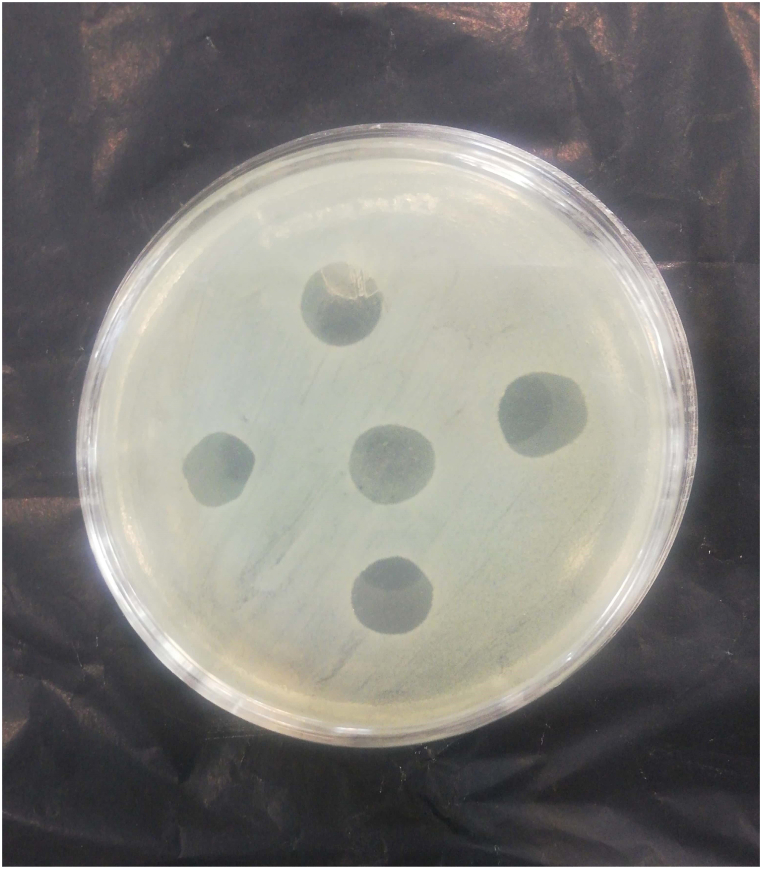
**Lysis of *Pseudomonas aeruginosa* PTCC 1074 with lytic bacteriophages in PEG ointment.***Pseudomonas aeruginosa* PTCC 1074 producing clear zone because of the bacteriophage in ointment. (The field of the plate includes the culture of *Pseudomonas aeruginosa* PTCC 1074, clear halos of five dilutions of PEG ointment after 24 h of adding bacteriophage to the PEG ointment were syringed onto the lawn's surface) PEG: polyethylene glycol.

Clear zones on the surface of lawn culture plates of standard *P. aeruginosa* were observed at different dilutions of PEG ointment containing bacteriophage. The most phage survival occurred 24 h after the ointment preparation (Fig. 3).

Table 2 exhibits pH alterations in PEG ointments (PEG 400/PEG 4000 = 8:4 (g) and PEG 400/PEG 4000 = 9:3 (g)) at varying temperatures for a period of four months. The data did not show any major disparities (*P* < 0.05) in pH modifications at various temperatures (4, 25, 40 °C).

**Table 2:** pH changes of polyethylene glycol (PEG) ointments at different temperatures.

	4 °C			25 °C			40 °C		
Month(s)	0	2	4	0	2	4	0	2	4
pH **(Ointment in ratio 2:1)** (W_PEG__4000_:W_PEG 400_)	4.95 ± 0.05	4.97 ± 0.04	5.03 ± 0.10	4.95 ± 0.05	5.01 ± 0.02	4.98 ± 0.07	4.95 ± 0.05	4.43 ± 0.02	4.01 ± 0.03
pH **(Ointment in ratio 3:1)** (W_PEG__4000_:W_PEG 400_)	5.01 ± 0.05	5.07 ± 0.04	5.20 ± 0.10	5.08 ± 0.05	5.04 ± 0.02	4.95 ± 0.06	5.10 ± 0.05	4.61 ± 0.03	4.28 ± 0.04

### 4.5 Animals

The baseline characteristics of mice are summarized in Table 3. Despite the insignificant statistical disparities between the groups in terms of burned wound size (*P* = 0.12), there were over 10% mean discrepancies in the mean quantities ascertained between the groups. Hence, the burned wound's size was considered a confounding factor. Following this factor adjustment, we did further analyzes.

**Table 3:** Mouse baseline characteristics of the different groups.

	Bacteriophage-treated group	Antibiotic-treated group	Polyethylene glycol group	Untreated- control group	*P-value*
Body temperature (°C)	36.2 ± 0.8	35.5 ± 0.7	36.9 ± 0.7	36.5 ± 0.8	**0.983**
Physical activity score	5.0 ± 0.0	5.0 ± 0.0	5.0 ± 0.0	5.0 ± 0.0	–
Burn wound's size (mm^2^)	112.0 ± 46.1	195 ± 74.4	140.5 ± 75.2	119.7 ± 25.2	**0.12**

The value are expressed as Mean ± SD, n = 6 in each group. *P-values* show comparison between the groups (one-way ANOVA test followed by Tukey's multiple comparisons *post-hoc* test). Significant variation considered at *P* < 0.05.

Five of the 12 deaths in the negative control (3 mice) and ointment-based (2 mice) group occurred within the first 24 h. Six mice per group (who survived 24 h) were obtained by replacing dead mice, allowing researchers to compare the effects of four intervention techniques on wound healing. Until the tenth day of the study, one more mouse in the antibiotic group and one more mouse in the negative control group suffered and died. In mice afflicted by burning/infection and treated with a daily topical application of phage, a 100% survival rate was noted compared to the untreated-control group which had a 50% survival rate in the first day post-wounding (*P* > 0.05). After the second day, the application of phage ointment on the burned and infected area on a daily basis yielded a substantially higher survival rate of 100%, versus the survival observed in the untreated-control group and antibiotic-treated group (83.3%) over the course of the ten days after the wounding.

After being treated with phage ointments, mice showed no signs of irritation. The skin of the treated mice was without inflammation or redness compared to the untreated.

### 4.6 Assessment of wound healing

Significant differences were found between the four topical treatment groups based on a repeated measure ANOVA statistical test (*F* (3,104) = 6.91, *P* < 0.001). The various time measurements did not yield significant differences (*F* (4,106) = 0.40). After Bonferroni correction, significant differences were found only when comparing the bacteriophage-treated group to the PEG-based ointment group. The untreated-control group showed a significant difference compared to the bacteriophage-treated group. The PEG-based group differed significantly from the antibiotic-treated group (*P* = 0.001, *P* = 0.001, *P* = 0.002, respectively).

**Fig. 4:**
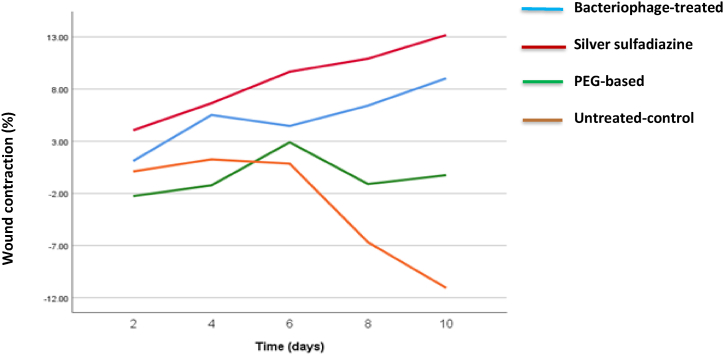
**Wound contraction in different wound dressings groups.** Wound contraction in untreated-control, antibiotic-treated, bacteriophage-treated, and PEG-based groups at different time points post injury. Data are expressed as means ± SD; n = 6. Statistically significant differences from control are marked by asterisks (*p < 0.05, **p < 0.01, ***p < 0.001; repeated measures ANOVA with Tukey's multiple comparisons *post-hoc* test).

The linear prediction trend line (Fig. 4) showed that the wound contraction rate increased over time more rapidly in silver sulfadiazine and PEG + Phges groups. In the other words, the % increment in wound contraction rate compared with baseline at all time points tending to be greater in silver sulfadiazine and PEG + Phges groups than in negative-control and PEG-based ointment groups. This trend was more clear in the last stages of post wounding day 8 and 10. The wound contraction in the PEG-based ointment group did not change significantly. With no treatment afforded to the untreated-control group, the burned wound deteriorated during the treatment course and the recovery of the wound skin could not be assessed.

The baseline wound area differed significantly between the groups, so it was normalized by considering the baseline as 100% for adjustment. The impact of group alone on the alteration in wound contraction was nearly statistically significant, as indicated by a repeated measure ANOVA (*F* (3,104) = 2.66, *P* = 0.052). However, the effect of time on the alteration in wound contraction was not significant (*F* (4,104) = 0.15, *P* = 0.96).

The mean wound's contraction during the treatment period was measured, and the results are shown in Table 4. Antibiotic-treated group had the largest wound area at baseline (195 ± 74.4 mm^2^). The wound contraction rate consistently increased over time in the antibiotic-treated group and almost in bacteriophage-treated group. The wound contraction rate was the smallest in untreated-control group on post-wounding day 8 and 10, but according to one -way ANOVA with Tukey's multiple comparisons *post-hoc* analysis there was no significant difference among the groups (antibiotic-treated group > bacteriophage-treated group > PEG-based group > untreated-control group, *P* = 0.59 and *P* = 0.42, respectively). The group treated with antibiotics had the smallest wound area on days 8 and 10, despite having the largest wound area at the beginning of the study.

**Table 4:** The wound contraction (%) changes.

	Day 2	Day 4	Day 6	Day 8	Day 10	*P-value*^a^	Adjusted *P-value*^c^
Bacteriophage-treated group	1.1 ± 0.9	5.5 ± 0.10	4.5 ± 4.13	6.4 ± 1.19	9.0 ± 1.20	0.92	0.88
Antibiotic-treated group	4.1 ± 4.10	6.6 ± 6.8	9.7 ± 7.8	10.9 ± 8.13	13.2 ± 9.12	0.68	0.71
PEG-based group	−2.3 ± 2.13	−1.2 ± 8.13	2.9 ± 1.19	−1.1 ± 8.30	−0.3 ± 8.36	1	1
Untreated- control group	0.1 ± 9.7	1.3 ± 3.10	0.8 ± 2.12	−6.7 ± 8.18	−11.1 ± 9.18	0.53	0.53
*P-value*^b^	0.76	0.60	0.78	0.59	0.42	-	–
Adjusted *P- value*^c^	0.71	0.58	0.76	0.67	0.48	-	–

The value are expressed as Mean ± SD, n = 6 in each group.

a *P*-values show time-point comparison of each group (repeated measures ANOVA).

b *P*-values show comparison between the groups (one-way ANOVA test followed by Tukey's multiple comparisons *post-hoc* test).

c Results were adjusted for baseline wound size.

As we mentioned before, the wound size on baseline was confounding factors because of over 10% mean differences between the groups (antibiotic-treated group > PEG-based group > untreated-control group > bacteriophage-treated group). Even after adjusting for wounds size as a confounding factor, Tukey's multiple comparisons *post-hoc* analysis showed no average difference between groups at each time point.

There were no significant differences in physical activity between treatment groups (*P =* 0.58) at baseline, as indicated by Fisher's exact statistical test. The physical activity of mice in the treated groups steadily increased during treatment and the variances were statistically significant (*P* < 0.001).

Changes in body temperature were observed over time and between groups, as presented in Table 5. The repeated measure ANOVA statistical test revealed no significant differences in body temperature among the four topically treated groups after adjusting for burned wound area as a confounding factor (*F* (3,104) = 1.71, *P* = 0.17). However, there were notable differences observed among the various time measurements (*F* (4,104) = 3.35, *P* = 0.007). After Bonferroni correction, the *post-hoc* comparison showed significant differences between day 8 and day 2, 4, and 6 (*P* = 0.001, *P* = 0.02, *P* = 0.02, respectively).

**Table 5:** Data on body temperature measurements in mice throughout the experiment.

	Day 2	Day 4	Day 6	Day 8	Day 10	P-value*	Adjusted *P-value*^b^
Bacteriophage-treated group	36.7 ± 0.7	36.0 ± 0.6	36.0 ± 0.7	37.5 ± 0.4	36.4 ± 0.6	0.003*	0.0001^b^
Antibiotic-treated group	36.0 ± 0.4	36.6 ± 0.5	36.3 ± 0.6	36.2 ± 0.8	35.9 ± 0.6	0.10	0.05^b^
PEG-based group	36.1 ± 0.6	36.3 ± 0.4	36.2 ± 0.6	36.8 ± 1.0	36.5 ± 0.8	0.23	0.23
Untreated- control group	35.8 ± 0.5	36.1 ± 0.7	35.9 ± 0.4	36.4 ± 0.5	36.4 ± 0.9	0.41	0.35
*P-value*^a^	0.05^a^	0.44	0.65	0.03^a^	0.61	_	_
Adjusted *P-value*^b^	0.07	0.63	0.27	0.10	0.75	_	_

The value are expressed as Mean ± SD, n = 6 in each group.* *P*-values show time-point comparison of each group (repeated measures ANOVA).

a *P*-values show comparison between the groups (one-way ANOVA test followed by Tukey's multiple comparisons *post-hoc* test).

b Results were adjusted for baseline wound size.

### 4.7 Histopathological findings

**Fig. 5:**
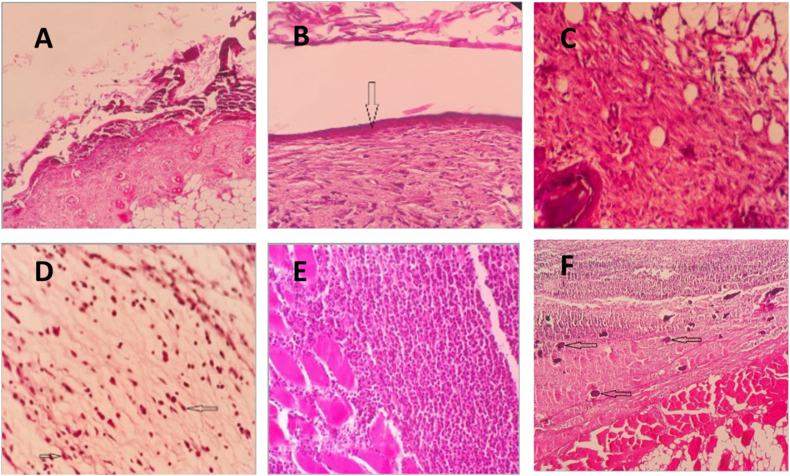
Hematoxylin/eosin staining (x100: A& B; x400: C–F) of the burns wounds site shows (A) skin ulcer covered by suppurative exudate and necrotic squamous cells (upper half) and granulation tissue formation in the dermis; (B) Early thin epithelialization (arrow), fibrotic granulation tissue in the dermis and sloughed keratin material in the top; (C) Mature fibrotic granulation tissue in hypodermis with the remnant of mature fat cells (lower right); (D) Immature granulation tissue comprises edematous stroma with few numbers of tissue culture–like fibroblasts (arrows), without significant collagen; (E) dense suppurative inflammation with an extension between necrotic striated muscle fibers (left third); (F) extensive necrosis in the epidermis and dermis replaced by suppurative exudate (upper half, foci of calcification (arrows), and necrotic skeletal (lower half and right)); on day ten post wounding.

Table 6 displays the histopathological findings of tissue samples from burned wounds. Qualitative data from histological evaluation on day ten, as seen in Table 6, has been represented in Fig. 5. While the antibiotic-treated and bacteriophage-treated groups had better wound healing parameters, the untreated-control group had the worst wound healing condition in all examined criteria (Table 6). Despite this, Fisher's exact test did not find any significant differences in the histopathological criteria.

**Table 6:** Histologic characteristics of the 10-day post-wounding.

Evaluated index^a^		Abundance					P-value*
	Score		Bacteriophage-treated group (N = 6)	Antibiotic-treated group (N = 5)	PEG-based group (N = 6)	Untreated- control group (N = 5)	
Granulation tissue coverage	Immature N(%)		0 (0)	0 (0)	4 (66.7)	2 (40.0)	0.08
	Mature N(%)		4 (66.7)	4 (80.0)	2 (33.3)	360.0	
	Fibrosis N(%)		2 (33.3)	1 (20.0)	0 (0)	0 (0)	
Granulation	Partially N(%)		1 (16.7)	1 (20.0)	2 (33.3)	4 (80.0)	0.07
	Thin N(%)		4 (66.7)	4 (80.0)	4 (66.7)	1 (20.0)	
	Thick N(%)		1 (16.7)	0 (0)	0 (0)	0 (0)	
Reepithelialization	Incomplete N(%)		3 (50.0)	2 (40.0)	4 (66.7)	5 (100.0)	0.33
	Complete, thin N(%)		2 (33.3)	3 (60.0)	2 (33.3)	0 (0)	
	Complete, natural N(%)		1 (16.7)	0 (0)	0 (0)	0 (0)	
Inflammation	Severe N(%)		3 (50.0)	3 (60.0)	2 (33.3)	3 (60.0)	0.88
	Moderate N(%)		2 (33.3)	2 (40.0)	4 (66.7)	2 (40.0)	
	Mild N(%)		1 (16.7)	0 (0)	0 (0)	0 (0)	
Tissue necrosis	Severe N(%)		2 (33.3)	1 (20.0)	4 (66.7)	4 (80.0)	0.21
	Moderate N(%)		4 (66.7)	4 (80.0)	2 (33.3)	1 (20.0)	
	Mild N(%)		0 (0)	0 (0)	0 (0)	0 (0)	

^a^Microscopic evaluated index degrees are:

Type of granulation tissue: immature, mature, fibrosis.

Granulation tissue score: partially, complete and thin, complete and thick.

Epithelialization: incomplete, complete and thin, complete and nearly normal.

Tissue inflammation: severe, moderate, mild.

Wound necrosis: severe, moderate, mild.

* *P*-values show comparison between the groups (Fisher's exact test).

## 5 Discussion

This paper is the first to examine PEG-based hydrophilic ointments containing bacteriophage on a murine model of infected burn wounds, as far as we know. According to the current study, semi-solid bacteriophage preparation can be used as a topical agent to promote the wound healing process, making it an appealing approach to treat burn wounds.

Severe life-threatening problems arise from post-burn infections, and a patient's survival depends on the severity of the burn and type of infection. Multidrug-resistant types of bacteria cause complex infections that most patients are infected with shortly after. *P. aeruginosa,* a pathogen that is intrinsically resistant to multiple drugs, is among the primary reasons for post-burn infections that are life-threatening [21].

Bacteriophages, or phages for short, are viruses that specifically target and infect bacteria. They have been studied for over a century as potential alternatives to antibiotics, especially in the face of the growing problem of antibiotic resistance. Recent advances in phage-based therapeutics have shown promising results and are providing insight into a possible post-antibiotic era [41].

Novel approaches to phage administration to patients are investigated. Using phage-containing sprays or gels that can be applied directly to the infection site is one method [41]. Several studies have evaluated the efficacy of topical bacteriophages in treating burn wounds, and the results have been promising [[42], [43], [44]]. There is also a study showed that the topically applied bacteriophage cocktail could reduce the bacterial load significantly in multi-drug resistant *P. aeruginosa* (MDR-PA) infected wounds in the New Zealand rabbit model, resulting in improved wound healing outcomes [45]. Following this, we isolated the lytic bacteriophages of *P. aeruginosa* and used them topically against this pathogenic bacterium. According to the above studies, we also found potential advantages of using bacteriophages over traditional antibiotics, such as their ability to target specific bacterial strains.

Our research is consistent with prior studies which presented promising outcomes regarding the use of bacteriophages to treat *P. aeruginosa*-infected burn wounds [[46], [47], [48], [49]]. Using bacteriophages as a potential treatment for bacterial infections has gained increasing attention in recent years. While phage therapy has been explored for over a century, recent advancements in molecular biology and genomics have reignited interest in this field [50]. The review article by Rostkowska et al. (2019) provides valuable insights into the history, current status, and future prospects of phage therapy. The article specifically highlights the potential benefits of using phage cocktails, which contain multiple phages with different host ranges and modes of action, to overcome the limitations of monotherapy. One of the key challenges of phage therapy is the emergence of phage-resistant bacteria, which can reduce the effectiveness of treatment. By using phage cocktail, it is possible to reduce the likelihood of resistance development, as well as increase treatment efficacy through synergistic effects.

Besides using PEG-based hydrophilic ointments for bacteriophage therapy, we also used a phage cocktail in our study. By using a combination of multiple phages with different host ranges and modes of action, we aimed to reduce the likelihood of resistance development and increase treatment efficacy through synergistic effects. Our findings suggest that the use of phage cocktail therapy, in combination with PEG-based hydrophilic ointments, has the potential to be a highly effective approach to treat infected burn wounds in murine models.

However, the use of phage cocktails also poses some challenges, such as the need for careful selection and characterization of phages, as well as the potential for immune response and toxicity. Our research showed that treating infected burn wounds in small animal models with a type of bacteriophage therapy that uses PEG-based hydrophilic ointments could be promising and might apply to human patients in the future. PEG may create sustained-release formulations by encapsulating phages in PEG-based matrices or by conjugating phages to PEG molecules [51]. Using PEG as a carrier for bacteriophages can improve their stability, bioavailability, and reduce their immunogenicity [52].

As previous studies have shown, our study showed that the topical phage formulation was just as effective as an FDA-approved product, topical silver sulfadiazine (Fig. 4). Examined the efficacy of topical treatment with phage kpn5 compared to the natural product aloe vera and honey [14]. Additionally, the investigators reported they tested the effectiveness of topical phage kpn5 formulation against topical gentamicin and silver nitrate [2]. Hydrogel-based phage kpn5 was found to be more effective in wound healing than natural or FDA-approved products. According to the present results, the phages included in the ointment resulted in the lysis of the host bacteria (Fig. 2). By entering the wound, the lytic phage could eliminate infections.

The self-replication and exponential growth of phages in the presence of the bacterial host enables them to exert bactericidal effects even at low concentrations [53]. Bacterial infection can only be controlled by a handful of phages [54], as our data determined a low effective bacteriophages concentration in wound healing. According to the study by Merabishvili et al., bacteriophage therapies need particles that are active and concentrated between 10^6^-10^7^ PFU/mL [55]. We determined 10^7^ PFU/mL as an effective therapeutic dosage, considering the high titer of bacteriophages in the body when in contact with suitable host bacteria.

We used bacteriophages in a topical ointment because reduced blood flow can impair delivery to the burn site. Topical delivery of antibacterial agents allows direct contact with wound surface [1]. Various topical formulations were studied by Chang and colleagues [56]. Phage preparation is traditionally applied to gauze after soaking it [56]. However, there are some challenges linked to this method, such as phages getting trapped in the gauze and incomplete phage release. Using spray devices is also an option, but there is a risk of phages getting stuck. Semi-solid forms such as gels, creams, or PEG ointment bases improved phage stability and overcame liquid formulation limitations. Phages could be carried effectively by semi-solid formulations, which can also hydrate and protect the skin. Applying these products is a breeze, cleaning them is easy, and they hardly cause any irritation [56]. Some phages may be inactivated by alcohol, so semi-solid water-based formulations are preferred over organic solvent-based ones. Phage release profiles are minimally affected by non-ionic vehicles [31, 57]. Non-ionic carriers were found to be more effective than anionic or cationic vehicles in terms of optimal recovery and lytic phage stability. The ionic nature, especially in the anionic-based carrier, could cause deactivation easily [58]. The phage-releasing profile could be affected by preservatives, as per another study [59].

Based on the studies mentioned above, we concluded that PEG ointments without added preservatives are the best carrier because of minimal interference with the phage structure and release process. To maximize its effectiveness without preservatives, we made our ointment daily. In ointments containing PEG, bacteriophages were active and stable (Fig. 2).

Our research revealed that the combination of PEG and bacteriophages applied to a *P. aeruginosa-*infected wound can help with healing and guard against further infection. Furthermore, application of topical phage reduces the chances of being removed by the immune system.

Mice that were given phage either immediately or 45 min after infection survived, as reported by Holguin et al. [60]. The wound did not completely heal because of the presence of bacteria in the lesion, leading to a skin mastocytosis immune response [60]. These findings were also confirmed by histopathology (Table 6). Treating mice either 24 or 48 h after infection resulted in effective healing of lesions [60]. The results prompted us to start treatment 24 h after infection induction.

The healing process of skin wounds in murine models involves secondary intention, granulation, and re-epithelialization, resulting in skin contraction [61,62]. On hematoxylin/eosin staining (Fig. 5), PEG + phage-treated cells showed better regeneration and reduced inflammation compared to the control cells. Then it would be reasonable to suggest that modulating cells involved in inflammatory and proliferative recovery phases through topical phage application may promote wound healing. Phages are known to mainly display anti-inflammatory effects and assist in immune system regulation. This provides hope for the application of phages in medicine beyond their commonly known antibacterial traits [37].

This study evaluated the safety and efficacy of a semi-solid phage preparation. Topical application of the treatment resulted in a 100% survival rate for mice with burned wound areas over a ten-day period, while the untreated control group had a survival rate of 83.3%. No irritation signs were observed in any of the groups, both 24 h after the experiment and for ten days. Even though we discovered that phages have therapeutic potential as a safe topical agent, it's doubtful that phage therapy will replace antibiotics; as a complementary treatment, it can be used. However, the remarkable source of bacteriophage extraction makes this approach a low-cost therapeutic option.

Topical bacteriophage therapy is still a valuable approach to antibacterial treatment for wound healing, even though the potential of advanced technologies in promoting wound healing and preventing infections is highlighted [[63], [64], [65]].

Semi-solid phage preparation and antibiotics could be combined to treat burn wounds, as suggested by the authors. Additionally, phages may contain antibiotic resistant mechanisms and virulence factors that can potentially be transmitted to bacteria via a hypothetical lysogenic cycle. To guarantee future use, sequencing of phages is necessary.

We may have limited the study by using only female mice. Some clinical/experimental literature shows the beneficial effect of female sex steroid hormones following trauma and suggests that survival outcomes are better in females than males for most injury types, including burn injury [[66], [67], [68], [69]]. Our data sets also found that female mice survive very well. It is therefore helpful to use female mice. Currently, no studies show the impact of the female estrous cycle on the injury types. Furthermore, we observed variability in wound contraction measurements. Wound healing is a complex process influenced by various factors, such as individual differences in healing rates, the extent and type of burn injury, and the influence of biological factors. These factors can contribute to significant variations in wound contraction, making it challenging to detect statistically significant differences, particularly with smaller sample sizes.

The significant findings between the four topical treatment groups and the greater histological healing suggest that the bacteriophage treatment may have a positive impact on the underlying tissue and promote favorable histological changes associated with wound healing. This finding is encouraging and supports the potential efficacy of the treatment in enhancing the healing process. However, we acknowledge that the large variation observed in wound contraction measurements may have limited our ability to detect significant differences between the various time measurements.

Additionally, the treatment duration in our study was 10 days, which is relatively short considering the complex nature of burn wound healing. We recognize that a more prolonged treatment regimen may yield more pronounced effects on wound contraction and other outcome measures. Future studies with extended treatment durations should be conducted to further investigate the treatment's long-term effects and provide a more comprehensive understanding of its potential benefits.

## 6 Conclusion

Despite the limitations associated with sample size and variability in wound contraction measurements, our study provides valuable insights into the effects of PEG-based hydrophilic ointments for bacteriophage therapy as a promising approach for treating infected burn wounds in murine models. This approach could also potentially be effective for treating human patients in the future. One potential advantage of using PEG as a carrier for bacteriophages is the ability to create sustained-release formulations. These findings highlight the potential of PEG-based hydrophilic ointments as a viable option for the development of bacteriophage-based therapies. Further studies are needed to fully explore the safety and efficacy of this approach, as well as to optimize the formulation and delivery methods for clinical use.

## Author contribution statement

Bibi Sedigheh Fazly Bazzaz: Conceived and designed the experiments; Analyzed and interpreted the data; Contributed reagents, materials, analysis tools or data; Wrote the manuscript.

Zhila Taherzadeh: Conceived and designed the experiments; Analyzed and interpreted the data; Contributed reagents, materials, analysis tools or data; Wrote the manuscript.

Hanieh Piranaghl: Performed the experiments; Wrote the manuscript.

Shiva Golmohammadzadeh: Analyzed and interpreted the data; Wrote the manuscript.

Vahid Soheili: Analyzed and interpreted the data; Wrote the manuscript.

Zahra Sabeti Noghabi: Conceived and designed the experiments; Performed the experiments; Contributed reagents, materials, analysis tools or data.

Bahram Memar: Analyzed and interpreted the data.

Seyede Melika Jalali: Performed the experiments.

## Data availability statement

Data will be made available on request.

## Additional information

No additional information is available for this paper.

## Funding

The Vice-Chancellor for Research of the Mashhad University of Medical Sciences, Mashhad, Iran, supported this work financially.

## Ethics approval

Ethical approval for this study was obtained from the Mashhad University of Medical Sciences Ethics Committee (ID: 960278).

## Declaration of competing interest

The authors declare that they have no known competing financial interests or personal relationships that could have appeared to influence the work reported in this paper.
